# What are the determinants of the willingness to share rides in pooled on-demand services?

**DOI:** 10.1007/s11116-020-10110-2

**Published:** 2020-05-14

**Authors:** María J. Alonso-González, Oded Cats, Niels van Oort, Sascha Hoogendoorn-Lanser, Serge Hoogendoorn

**Affiliations:** 1 Department Transport and Planning, Civil Engineering and Geosciences, Delft University of Technology, P.O. Box 5048, 2600 GA Delft, The Netherlands; 2 KiM Netherlands Institute for Transport Policy Analysis, Postbus 20901, 2500 EX The Hague, The Netherlands

**Keywords:** Willingness to share, Ridesharing, DRT, Stated preference, Choice modelling, Scenario analysis

## Abstract

Simulation studies suggest that pooled on-demand services (also referred to as Demand Responsive Transport, ridesharing, shared ride-hailing or shared ridesourcing services) have the potential to bring large benefits to urban areas while inducing limited time losses for their users. However, in reality, the large majority of users request individual rides (and not pooled rides) in existing on-demand services, leading to increases in motorised vehicle miles travelled. In this study, we investigate to what extent fare discounts, additional travel time, and the (un)willingness to share the ride with (different numbers of) other passengers play a role in the decision of individuals to share rides. To this end, we design a stated preference study targeting Dutch urban individuals. In our research, we (1) disentangle the sharing aspect from related time–cost trade-offs (e.g. detours), (2) investigate preference heterogeneity regarding the studied attributes and identify distinct market segments, and (3) simulate scenarios to understand the impact of the obtained parameters in the breakdown between individual and pooled services. We find that less than one third of respondents have strong preferences against sharing their rides. Also, we find that different market segments vary not only in their values of the willingness to share, but also in how they perceive this willingness to share (per-ride or proportional to the in-vehicle time). Further, the scenario analysis demonstrates that the share of individuals who are willing to share rides depends primarily on the time–cost trade-offs, rather than on the disutility stemming from pooling rides per se.

## Introduction

The new on-demand mobility services appearing in cities can foster a shift from the current ownership paradigm into a service paradigm (ITF 2017). Among these services, the uptake of on-demand rides (provided by companies such as Uber, Lyft, DiDi Chuxing or Grab and also known as ridesourcing or ride-hailing services) has been remarkable all over the world, with Uber alone serving 14 million trips a day (Uber 2019).

On-demand rides improve their users’ accessibility, given that (a) these individuals are less likely to own a car themselves (Rayle et al. 2016), and (b) these services are often used for trips that would have taken over twice as long by public transport (Rayle et al. 2016). However, recent research has shown that these services have also increased motorised vehicle miles travelled (VMT) (Erhardt et al. 2019; Henao and Marshall 2018) due to empty vehicle miles, induced trips, and modal shifts from public transport, cycling and walking. This increase in VMT has also been acknowledged by the on-demand providers themselves (Hawkins 2019).

On-demand ride requests can be categorised as individual or pooled, depending on whether the user is willing to share her ride with other passengers for a cheaper fare. The increase in VMT stems from the fact that, to date, the large majority of on-demand trips are individual trips. Currently, on-demand providers do not always offer pooled rides, and even in cities where pooled alternatives are available, only 20% of on-demand users request pooled rides (Gehrke et al. 2018), amounting to around 20% of the rides (Chen et al. 2018; Uber 2018). Further, the share of rides that has been eventually pooled together in on-demand operations with at least one other ride for part of their trip has been found to be much lower [around 2% in Denver (Henao and Marshall 2018) and 6–7% in Chengdu (Li et al. 2019)]. Tachet et al. (2017) found that the potential of sharing trips in very diverse urban settings in massive, but the current density of requested pooled on-demand trips seems too low to enable that.

Pooled rides (also known as ridesharing or ridesplitting) can help achieve large benefits regarding traffic, emissions, accessibility and parking in urban settings compared to the current situation (ITF 2016, 2017). However, benefits of pooled rides will only materialise if enough individuals are willing to adopt them. While Fagnant and Kockelman (2018) estimate that a market share of pooled services of 20–50% would be necessary to bring tangible mobility improvements, results from Rodier et al. (2016) indicate that even a higher share is likely to be necessary. In the San Francisco context, Rodier et al. show that if participation is as low as 20%, less than 1% of the trips end up being sharable, with negligible VMT reductions. They find that a participation rate of at least 50% is necessary to achieve large VMT reductions from pooled services. But, are individuals willing to share their rides?

After analysing the characteristics of over one million on-demand trips using an ensemble learning model, Chen et al. (2017) identified in-vehicle travel time and trip cost as the most important determinants of whether an individual will choose to share or not their trip with other passengers. In their model, time and cost attributes had importance weights of over twice as much as the other studied attributes (e.g., pick-up time or weather). Additional service attributes were investigated in Al-Ayyash et al. (2016), who used stated preference data. Al-Ayyash et al. (2016) found that the number of co-riders that can be assigned per vehicle is also an important factor regarding the willingness to adopt a pooled trip. The percentage of individuals willing to use the presented pooled on-demand service was 7–8% higher if the trip could be shared with a maximum of two additional passengers rather than if it could be shared with up to five additional passengers. This leads us to our central research questions: What is the value of time (VOT) (time–cost trade-off) of individuals for on-demand rides? And, what is the monetary disutility associated with sharing an on-demand ride with (different numbers of) other passengers (denominated hereby as the willingness to share (WTS))?

To answer these questions, our methodology approach comprises the design and analysis of a stated preference experiment. We include in-vehicle time, cost and the number of additional passengers as mode attributes. Given that on-demand providers can cater for different market segments by offering a variety of services, it is valuable to understand taste variation of individuals regarding the studied attributes. We account for both continuous and discrete taste heterogeneity in our model estimation. Additionally, we simulate different scenarios to better understand the impact of different time–cost trade-offs (and the impact of different numbers of passengers) on the breakdown between an individual and a shared alternative, based on the estimated parameters of our choice model analysis. We target individuals living in urban areas of the Netherlands in our research.

We summarise the aims of the current research as follows:

- Quantify the WTS in on-demand services for different numbers of passengers and the VOT, in order to disentangle the sharing aspect from related time–cost considerations (e.g. detours) in the context of choosing between individual and pooled rides.
- Analyse preference heterogeneity regarding the WTS and VOT for these on-demand services, and whether distinct market segments can be identified.
- Simulate the effect that different price–cost trade-offs and that different number of co-riders have on the breakdown between an individual and a pooled alternative based on the estimated parameters. We do so by means of a scenario analysis varying the previously mentioned attributes.

The rest of the paper is structured as follows. First, we review previous research regarding the factors that play a role in choosing a pooled on-demand ride over an individual alternative. Then, we introduce the research methodology, which includes the survey design, data collection, discrete choice analysis and scenario design. The subsequent results section presents the choice modelling results and the scenario analysis (as model application). Last, the discussion section provides further interpretation of the study findings, before the main conclusions are drawn.

## Literature review

In this section, we review previous literature that tries to understand individuals’ decision to share rides. This decision is of utmost importance in dense urban settings, given that simulation studies have shown that, had the majority of the on-demand trips been pooled, they would have reduced both the VMT and the number of required vehicles, even when taking into account the extra distance due to the involved detours (Bischoff et al. 2017; Rodier et al. 2016; Sun and Zhang 2018; Tirachini and Gomez-Lobo 2019). And pooling rides will become even more relevant in the age of autonomous vehicles, when riding on-demand services and not driving one’s own car may become the rule.

Three main attributes play a role in the decision of individuals to choose for a pooled ride over an individual alternative: the fare discount, the additional travel time incurred, and the (un)willingness to share the ride with other passengers. Time, cost and the number of co-riders are the aspects investigated in this study, and, therefore, are the focus of this literature review. Still, it is worth mentioning that there are other motives that can impact individuals’ decision to adopt on-demand services (see Tirachini (2019) for a recent overview), and that, other than the mere utilitarian motives, adoption of shared mobility in general has been linked to environmental and social aspects (Ciasullo et al. 2018; Javid et al. 2017; Mattia et al. 2019; Min et al. 2019).

The cost and time trade-offs that individuals encounter when choosing between the individual and pooled alternatives can be measured in monetary units versus minutes. Bösch et al. (2017) estimated that pooled on-demand services in urban settings can imply a cost benefit of 30–40% versus the individual alternative, whereas currently offered savings vary between 25 and 60% (Shaheen and Cohen 2018). Regarding time loss, empirical research has found that individuals experience (on average) ten minutes of added travel time as a result of pooling rides (Li et al. 2019). But simulation studies have shown that there is potential to reduce this value substantially. Previous research has shown that an average time disutility of less than three minutes per passenger is possible if all New York (Alonso-Mora et al. 2017) or Berlin (Bischoff et al. 2017) taxi rides would be requested as pooled rides. And Sun and Zhang (2018) estimated a travel time increase of 25% as a result of pooling rides. More generally, Tachet et al. (2017) has found that less than five minutes delay per passenger can provide successful matching potential in very diverse urban settings (e.g., for the Amsterdam context, a request trip density of 2.5 trips/h/km^2^ would already enable a matching rate of 92%).

The willingness to share (WTS) is more difficult to quantify. Sarriera et al. (2017) indicate that safety concerns, feelings of prejudice and the fear of having negative social interactions may deter individuals from requesting pooled rides. But the question is, to what extent? One way to measure part of this WTS aspect is to monetize how much money individuals are willing to pay to (not) share their trip with other individuals. We identify seven relevant stated preference studies that quantify the effect of sharing the ride with other individuals (see Table 1). The WTS is modelled in these studies either by a mode specific parameter (the alternative specific constant, ASC), which captures the difference of that alternative from all the other presented alternatives, or by an attribute of one of the included alternatives. The effect of additional passengers is measured either by a fixed number, by a range of fellow passengers (depending on vehicle capacity), or by the number of additional pick-ups (hence assuming that the pooling disutility is a function of the extra stops during the ride and not the number of extra passengers in the vehicle).

**Table 1:** Characteristics of SP studies with the willingness to share attributes

Study	Target population and setting	Mode alternatives*	Attributes considered				Mobility patterns and socioeconomics that play a role in the likelihood of individuals to…		Scenario analysis included?
			Time	Cost	Willingness to share	Number of extra pass	…shift towards pooled on-demand rides from current used mode	…prefer pooled on-demand rides over individual on-demand rides	
Al-Ayyash et al. (2016)	University students commuting by car or PT (Beirut, Lebanon)	Shared taxi, current mode (car or PT)	X	X	–	X (1–2 or 3–5)	Commuting mode (car or PT), gender	–	Yes, adoption rate of the new shared taxi service (versus current PT and car shares) with varying waiting time, in-vehicle time, trip cost and maximum number of additional passengers
Chavis and Gayah (2017)	Commuters (commuting trip) (Baltimore, US)	Bus, microtransit, individual taxi	X	X	X (ASC)	–	–	Car is primary mode, age	No
Krueger et al. (2016)	Residents (major Australian metropolitan areas)	Individual SAV, pooled SAV, current mode	X	X	X (ASC)	–	Car usage (as passenger or driver), multimodality degree, car-sharing usage, trip purpose	–	No
Lavieri and Bhat (2019)	Commuters (commuting and leisure trips) (DFW Metropolitan Area, US)	Individual SAV, pooled SAV	X	X	X (ASC)	X (1, 2, or 3)	Commuting mode, age, urbanity degree, ethnicity, working situation, income, household composition	Gender, ethnicity, education, income, vehicle availability	No
Liu et al. (2018)	Residents (New York, US)	Uber, UberPool, current mode	X	X	X (ASC)	–	–	–	Yes, impact of varying fleet sizes and impact of a per-ride tax
Steck et al. (2018)	Commuters (commuting trips) (Germany)	Walk, bicycle, AV, SAV (individual or pooled), PT	X	X	X (Add. pass.: yes/no)	–	–	–	No
Yan et al. (2018)	University students and staff (Michigan, US)	Car, flexible pooled transit, bicycle, walk	X	–	–	(X) Add. pick-ups (0, 1, or 2)	–	–	Yes, forecast of the modal share of the new flexible pooled transit (which replaces current bus service) under different deployment scenarios

^*^PT public transport, *AV* (privately owned) autonomous vehicle, *SAV* shared autonomous vehicle

The WTS parameters’ values were found significantly different from zero (i.e. the null hypothesis) in previous studies (except for Steck et al. 2018). The magnitude of their impact remains, however, inconclusive. As indicated in Table 1, the studies were conducted in different geographical contexts. This can presumably partly explain the differences observed. Next to cultural differences, differences can stem from differences in the familiarity with on-demand services or in public transport usage (i.e., familiarity with collective transport modes). Finally, the survey design can also play a role in the outcome. For example, while Lavieri and Bhat (2019) found that cost-time trade-offs are much more relevant than the sharing disutility itself, Krueger et al. (2016) found that the relevance that individuals attach to sharing, makes them perceive individual and pooled services as two distinct mobility options rather than an extra disutility resulting from sharing the ride. Individuals in the first study are familiar with on-demand services thanks to the current popularity of Uber and Lyft in the USA, and these operators offer similar services for both individual and pooled alternatives (other than the differences in time and cost). On the other hand, Krueger’s respondents may have been less familiar with pooled services (data collection took place earlier on and on-demand alternatives were not as prevalent in the Australian context). This may lead their respondents to consider both services as different alternatives altogether. Other than whether the trip is shared or not, the number of additional passengers with whom the ride is shared also has an influence on individuals’ preference. Alternatives with fewer passengers (or pick-ups) are preferred.

Some of the mentioned studies have also identified certain socioeconomic characteristics, mobility patterns and trip purposes that impact the willingness of individuals to shift towards pooled on-demand rides from their current mode and/or to prefer pooled on-demand rides over individual on-demand rides. For example, Lavieri and Bhat (2019) found that young individuals are more likely to adopt both individual and pooled services than older individuals, and Chavis and Gayah (2017) found that individuals younger than 25 years old seem to prefer pooled rides over individual rides. Pertaining to other socioeconomic characteristics, Lavieri and Bhat (2019) also found that the likelihood to adopt pooled services is lower for non-Hispanic whites, full time and self-employed workers, high income individuals and among those living alone.

Regarding mobility habits, more multimodal individuals (Krueger et al. 2016) and those who do not commute by car (Lavieri and Bhat 2019) are more likely to adopt on-demand rides (both individual and pooled). And those individuals that have the car as main transport mode tend to prefer individual rides over shared alternatives (Chavis and Gayah, 2017). In order to increase the likelihood of car users to shift towards pooled on-demand services, a good level of service should be provided. Al-Ayyash et al. (2016) identified level of service as the main factor for this group of individuals, while cost was the most important determinant for public transport users. Also, having used car-sharing schemes seems to increase the likelihood to adopt pooled services (Krueger et al. 2016).

Finally, trip purpose was also found to be an important determinant. Krueger et al. (2016) found that pooled rides were preferred over individual rides for shopping trips. Also, Lavieri and Bhat (2019) found differences in the characteristics of individuals interested in pooled rides depending on their trip purpose. They found that females, young individuals and those who had a car in the household were less likely to prefer the pooled alternative for commuting trip purpose, while highly educated individuals were less likely to prefer the pooled alternative for leisure trip purposes.

Leveraging on the estimated behavioural models, three of the stated preference studies included in Table 1 also include a scenario analysis. Al-Ayyash et al. (2016) and Yan et al. (2018) show how the predicted shares of their pooled on-demand alternative would decrease in scenarios where more additional passengers/pick-ups are expected. These studies, however, do not include an individual alternative. Thus, a comparison between potential individual and pooled shares is not possible. Liu et al. (2018), on the other hand, does consider both individual and pooled on-demand services in their model. They show different forecasted modal shares for these services with varying fleet sizes and the impact of a per-ride tax. They study these scenarios from a service design perspective, with the objective of optimising the supply-side parameters.

Our research adds to the aforementioned studies in two ways. First, it delves further into the characteristics that underlie the heterogeneity in individuals’ WTS and identifies different market segments. Previous studies account for taste variation related to different characteristics rather than identifying different groups. Second, it provides a scenario analysis of the impact of different time–cost trade-offs taking into account both individuals’ WTS and the disutility associated with having different numbers of additional passengers. Previously, these two attributes had only been modelled together in Lavieri and Bhat (2019), without offering a scenario analysis. Contrary to Liu et al. (2018), our scenarios aim to provide insight into the time–cost trade-offs of individuals and the effect of varying numbers of passengers, instead of the effect that different fleet sizes have on the overall modal share of the on-demand system or the effect that additional external costs have on system profitability.

## Methodology

The methodology section consists of four parts: survey design, data collection, discrete choice modelling methodology and scenario design.

### Survey design

**Fig. 1:**
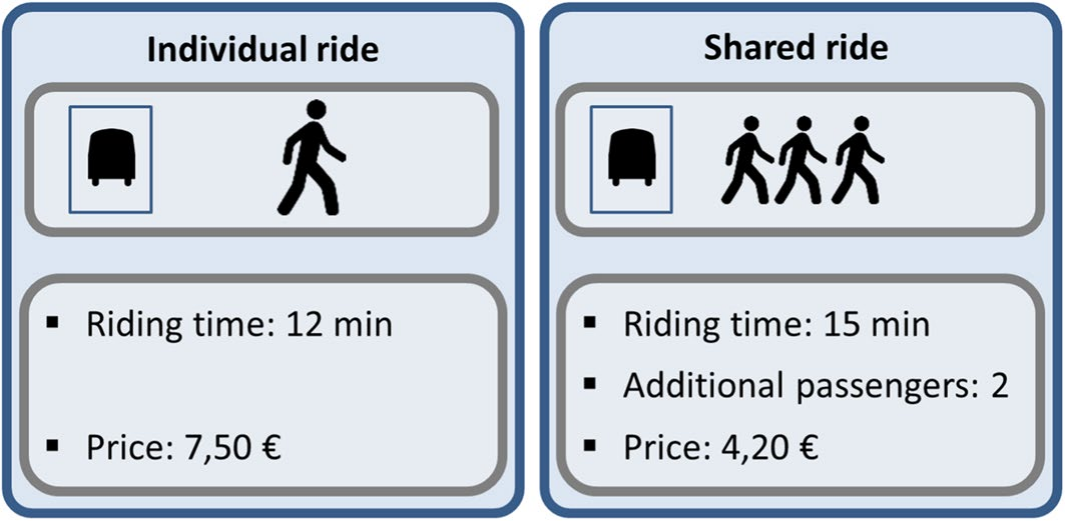
Example of a scenario of the stated preference experiment

To quantify the willingness to share rides in on-demand services, we design a Stated Preference (SP) experiment. SP experiments present respondents with hypothetical situations and have been widely used in the transport literature to obtain behavioural information in scenarios that differ from the status-quo. Unlike in the USA or China, there are, at the time of writing, no large scale pooled on-demand services in the Netherlands. Thus, obtaining revealed preference data for our research purpose is not possible. We opt for a labelled experiment with two alternatives (individual ride or shared ride). We include in-vehicle time, trip cost, and the number of additional passengers of the pooled alternative as SP attributes. Figure 1 shows an example of a choice task. The SP setting is either a commuting trip (shown to 70% of the working respondents who do not require their own private car for their commute and have commutes of at least 2 km) or a leisure trip (shown to the remaining respondents).

**Fig. 2:**
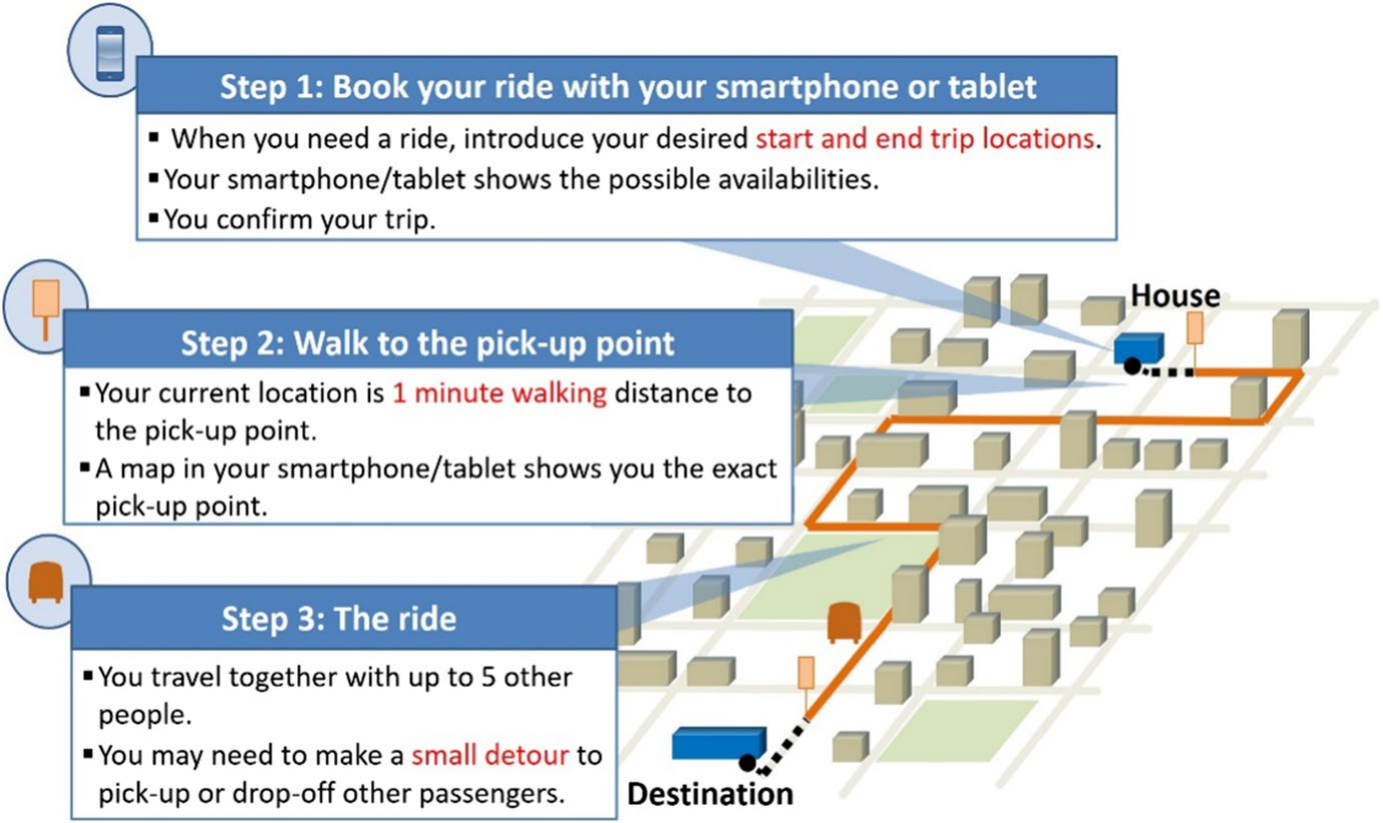
Included description of pooled on-demand services. Layout inspired by Kim et al. (2017). Small adjustments were made to this representation for individuals with no 3G connection and to individuals with traditional mobile phones (as opposed to smartphones). Individuals with a smartphone but no 3G/4G data bundles were additionally offered trip updates via sms, whereas individuals with a mobile phone but no smartphone were offered to make their bookings by means of a phone call and received the exact pick-up address via sms

This SP experiment is the last part of a more extensive survey focused on pooled on-demand services (which also includes a value of reliability study and attitudinal statements). The individual alternative is introduced to respondents for the first time during this SP experiment. Pooled on-demand services are presented to respondents at the beginning of the survey as depicted in Fig. 2. To increase realism, the individual alternative is constrained to be always quicker (non-existent high occupancy vehicle lanes in our context) and more expensive than the pooled option.

The experimental design of the SP experiment is an orthogonal fractional factorial design with blocking. Orthogonal designs offer robust parameters and do not require reliable priors (in contrast to efficient designs) (Walker et al. 2018). We also add its foldover design. The foldover design is the mirrored original design. It doubles the number of scenarios with the aim of obtaining uncorrelated two-way interactions of the attributes. We decide to add the foldover design given that the disutility to have extra additional passengers may be correlated with the time and/or cost attributes (see Metrics (2012) for more information regarding experimental SP designs). Our complete SP design results in six blocks with four scenarios each. For the attribute levels, we consider two set of values, depending on the length of the respondent’s reference trip (≤ 12 km or > 12 km), following the approach used in Arentze and Molin (2013). Attribute levels for time and cost for both versions are chosen such that similar values of time could be obtained in the model estimation. Attribute levels are depicted in Table 2.

**Table 2:** Attribute levels of the SP experiment depending on the length of the respondents’ reference trip

	Short trip SP version			Medium trip SP version		
	Level 1	Level 2	Level 3	Level 1	Level 2	Level 3
Expected time (Individual ride) [min]	10	15	18	20	25	28
Extra expected time (Shared ride) [min]	3	6	9	4	7	12
Cost (Shared ride) [€]	2	4	6	3	5	7
Extra cost (Individual ride) [€]	0.5	2.2	3	0.6	2.2	3
Number of other additional passengers (Shared ride) [add. passenger]	1	2	4	1	2	4

Travel choices can be influenced by attitudes (Domarchi et al. 2008). Therefore, in addition to the SP experiment, we include a series of 5-point Likert scale attitudinal indicators. They cover attitudes towards the three attributes included in the SP experiment (privacy, cost, and time) and serve two aims in our study: (1) understand if respondents’ differences in preferences towards individual and pooled services stem from different time–cost attitudes and/or differences in privacy attitudes, and (2) understand the main cause underlying non-trading behaviour, which could stem from either strong preferences for a particular alternative (for which the offered trade-offs are insufficient to result with a modal switch) or non-utility maximising behaviours (e.g., fatigue or boredom). We refer the interested reader to Hess et al. (2010) for more information on non-trading behaviour.

### Data collection

The survey was distributed on-line on May 2018 (in Dutch). Prior, initial modelling of an on-line pilot performed on April 2018 validated that the chosen SP attribute levels were adequate for our modelling purposes. Target respondents were individuals aged 18 years and older with a mobile phone living in highly urbanised areas in the Netherlands [defined as areas with more than 1500 inhabitants/km^2^ (Centraal Bureau voor de Statistiek (CBS) 1992)]. Survey respondents were recruited from a household panel designed for the longitudinal study of travel behaviour in the Netherlands: the Netherlands Mobility Panel (MPN) (Hoogendoorn-Lanser et al. 2015). This provided access to information on socioeconomic and mobility characteristics of respondents. All individuals invited to fill in the survey of this study belonged to different households.

### Discrete choice modelling methodology

We analyse the SP experiment using discrete choice analysis, under the Random Utility Maximisation (RUM) framework (Train 2003). We test different model specifications in our analysis, including interactions of the sharing attribute with the cost and time attributes (i.e., we test whether the disutility of sharing the ride with other passengers is a per ride disutility or it increases with increases in trip duration or trip costs). We also test whether different socioeconomic characteristics help explaining individual preferences for either of the two alternatives (and, if significant, quantify what their impact is), as well as whether both trip purposes are best modelled separately or jointly.

Our first model is a multinomial logit model with an added panel component to account for the correlations among the different observations of the same individual (making it a mixed logit (ML) model). A shortcoming of this model is its inability to account for unobserved heterogeneity, which is exclusively incorporated into the error term. Two main approaches exist to improve the specification in this respect: accommodate heterogeneity in certain continuous distributions of the modelled parameters (using more complex mixed logit (ML) models) or account for heterogeneity by identifying a discrete number of distinct classes, each having different (discrete) values for the modelled parameters (using latent class choice models (LCCM)). In other words, the first approach considers that the unknown parameters are randomly distributed over the population following a certain distribution instead of having a unique value. Alternatively, the second approach considers individuals’ heterogeneity by allocating them to different classes in a probabilistic fashion.

Both ML and LCCM have strengths and weaknesses (Greene and Hensher 2003; Hess 2014). To make the most of the strengths of both modelling approaches, we include a ML model with random coefficients and a LCCM in our analysis. The first one is able to encompass the overall heterogeneity of the data with a reduced number of parameters. The second one provides flexibility to define different attribute specifications for different classes, as well as is able to link taste heterogeneity to sociodemographic indicators. We use this second model to identify different market segments regarding pooled on-demand services. We refer the reader to Hess (2014) and Walker and Ben-Akiva (2002) for more information on these model structures and their mathematical specification.

In our analyses, we use 80% of our sample for modelling, and keep the remaining 20% for validation [as was done in Atasoy et al. (2010)]. We use the software PythonBiogeme (Bierlaire 2016) for modelling the ML models, and make use of the dedicated latent class software LatentGOLD (version 5.1) (Vermunt and Magidson 2016) for the LCCM analysis.

### Scenario design

To better understand the impacts of the time–cost trade-offs involved in the WTS rides in on-demand services, we used the estimated discrete choice models to perform a scenario analysis. We are interested in studying a wide range of trade-offs, and we use the widest trade-offs allowed given the design of the experiment, since the validity of the estimated parameters cannot be extrapolated beyond the range of values used for their estimation. This allows us to estimate scenarios with an excess of 3–12 min in in-vehicle time for the pooled alternative (compared to the individual option). This range covers both the minimum mean added time that simulation studies have reported (3 min Alonso-Mora et al. 2017; Bischoff et al. 2017) as well as the average added time found in empirical studies (10 min Li et al. 2019). Regarding price difference between both services, Bösch et al. (2017) estimated that pooled services can reduce individual prices on 30–40% in urban settings. Currently, savings of 25–60% for the pooled alternative can be expected (Shaheen and Cohen 2018), with prices for Dutch pooled options ranging between €3.50 (BrengFlex in the Arnhem-Nijmegen area) and €5.00 (ViaVan in Amsterdam). We cover a larger range of values in our scenarios (within the range of values included in the survey).

To ease the scenario comparison, we design a base scenario with the following characteristics: 20 min (mean time for single rides (Li et al. 2019)) and €6.00 for the individual ride, and + 7 min and €−2.00 (− 33%) for the pooled ride. Scenarios are computed using Monte Carlo simulation (100,000 draws used). The full sample (and not just the 80% used for estimation of the parameters) is used.

## Results

We divide the results section into three sections. First, we depict the descriptive analysis, including the description of the data collection and sample and an evaluation of the non-trading behaviour; then, we cover the choice modelling analysis; and the last part reports the scenario analysis, as final model application.

### Sample description and descriptive analysis

A total of 1077 respondents finished the questionnaire, of which 1006 (93%) were considered valid after data cleaning (based on survey completion time and straight lining checks throughout the whole survey). Table 3 shows the socioeconomic characteristics of the sample, the target population (highly urbanised areas in the Netherlands), and the overall Dutch values. Gender and the two urbanisation levels are well represented in our sample. Sample age distribution is overall representative of the respective population, although middle aged adults are a bit underrepresented and the elderly population slightly overrepresented. Shares for education, working status and household composition can only be compared to the national values. As expected, our (sub)urban sample has a higher percentage of higher educated individuals, working respondents and single households. Given the similitudes between the analysed sample shares and their Dutch counterparts, we consider that our sample adequately mirrors the socioeconomic characteristics of the target population.

**Table 3:** Comparison between the survey sample and the Dutch population. Sources for the population data: (Centraal Bureau voor de Statistiek (CBS) 2018a), (Centraal Bureau voor de Statistiek (CBS) 2018b)**,** (Centraal Bureau voor de Statistiek (CBS) 2018c)**,** (Centraal Bureau voor de Statistiek (CBS) 2018d)

Socio-economic variable	Category	Total sample (N = 1006) (%)	Dutch (very) high urbanised areas (%)	Dutch 2018 shares (%)
Gender	Male	48.2	48.9	49.6
	Female	51.8	51.1	50.4
Age	18* to 39	38.1	38.1	31.8
	40 to 64	35.6	42.0	44.0
	65 and above	26.3	19.8	24.2
Education**	Low	25.2		31.5
	Medium	32.5		37.8
	High	42.0		29.2
	Unknown	0.2		1.4
Work status	Working	59.9		50.9
	Not working	40.1		49.1
Household	1 person household	49.0		38.2
	> 1 person household	51.0		61.8
Urbanisation level	> 2500 inhab./km^2^	46.9	48.2	23.3
	1500–2500 inhab./km^2^	53.1	51.8	25.1

*18 to 39 for the share sample, but 20 to 39 for the Dutch population 2018 values

**Low: no education, basic education or uncompleted general secondary education; Medium: completed general secondary education (diploma to be admitted to higher education attained); High: bachelor candidate or above at a university or university of applied sciences

Out of the 1006 respondents, 308 were directed to the commuting trip purpose and 698 answered the survey for the leisure trip. The leisure trip purpose subsample had 42% of working individuals. Differences in working status between both subsamples led to differences in age and education levels (higher proportion of older and lower-education level individuals in the leisure subsample).

A significant share of respondents (around 30%) exhibited a non-trading behaviour in the SP experiment, despite that all blocks contained scenarios with values of time that ranged from less than 5 €/h to over 30 €/h (initial choice modelling analysis showed an average value of time of around 15 €/h). 50% of non-traders chose the individual alternative in all of the shown scenarios (we refer to these respondents as “individual-only” respondents), and the other 50% chose exclusively the pooled alternative (“pooled-only” respondents). Given the link between attitudes and behaviour (Molin et al. 2016), we perform an exploratory factor analysis (EFA) on the included privacy, cost and time related attitudinal indicators to shed light on the main reason behind the exhibited non-trading behaviour. We use principal axis factoring with direct oblimin rotation, and extract factors with eigenvalues greater than one (Kaiser–Meyer–Olkin measure KMO = 0.797 and Bartlett’s test of sphericity *p* < 0.001, indicating sampling adequacy and adequate correlation between the EFA items). The included statements and the related performed analysis is included in “Appendix A”.

We extract three factors from the EFA (privacy, cost and time factors), as expected. We measure the reliability of the factors with the Cronbach’s alpha coefficient, and obtain (for the Cronbach’s Alpha based on the standardized items) 0.61 (privacy factor), 0.70 (cost factor) and 0.57 (time factor). Values over 0.60 are considered acceptable in exploratory research (Nunnally and Bernstein 1994). Cronbach’s Alpha value, however, is dependent on the number of items that belong to a factor (Tavakol and Dennick 2011), which explains the somewhat lower value for the time factor (which consists of two items). Following Schmitt (1996) and Taber (2017), which argue that factors with lower alphas can also prove both acceptable and useful, and after checking that the two attitudinal time items are highly correlated (their Pearson correlation is 0.40), we decide to not discard the time factor due to the exploratory (and not confirmatory) nature of our factor analysis.

The means of all attitudinal indicators display the same trend: “individual-only” respondents (15% of the sample) are the most privacy and time sensitive, and the least cost sensitive; the opposite holds for the “pooled-only” respondents (15%). The mean values of “traders” (70%) lie always in between both two groups. ANOVA tests confirm that these differences are significant for all indicators at the 95% confidence level or beyond. This difference is largest between the “individual-only” and the “pooled-only” groups, significant at the 99% level (independent t-test). Therefore, we consider the existence of strong preferences as the main underlying cause for the non-trading behaviour, and accept non-traders as valid respondents in the posterior choice modelling analysis.

Further, pair-wise comparison between “individual-only” and “traders” shows statistically different means in all indicators (in all but one at the 99% level) while differences between “pooled-only” and “traders” are insignificant for some of the privacy indicators. This suggests that differences in preferences between “individual-only” and “traders” stem from both different values of time and willingness to share, while differences between “pooled-only” and “traders” stem mainly from differences in the values of time of the two groups.

### Discrete choice model estimation

We estimate three model structures (see Table 4), as indicated in the Discrete Choice Modelling Methodology subsection. The first model is a mixed logit model with a random component to account for the panel structure of the data. All included parameters in this first model are significant and have the expected signs. Time and cost are modelled linearly as generic parameters (i.e., they have the same parameters for both alternatives). We find that working individuals have a larger time disutility, and include this taste heterogeneity in the model with an additional time disutility parameter for this segment of the population. The models tested show that the effect of the number of additional passengers is best modelled as a trip specific disutility for the case of one or two extra passengers (same disutility for both situations). However, the WTS disutility for the four extra passengers scenario is higher (starting at 20% higher for 13 min rides, the shortest trip included in the experiment) and increases per minute of in-vehicle time. We speculate that individuals consider that a similar level of privacy and enough personal space is granted in both the single and the two co-rider scenarios, which may explain why the same disutility is attributed to both scenarios. This threshold is however surpassed for the four co-rider situation, leading not only to a higher value but to a per-minute value. We find that having a high income, never using bus/tram/metro (BTM) and having a low usage of cycling increases the preference towards the individual ride alternative. These effects are also included in the model specification. We also find, that, unlike in Lavieri and Bhat (2019), commuting and leisure trip purposes are best modelled together [tested using a likelihood ratio test (Ben-Akiva and Lerman 1985)].

**Table 4:** Parameter values (and robust t-tests) of the mixed logit (ML) models and parameter values (and z-value) of the latent class choice model (LCCM) (p-value: ≤ 0.01 ***, ≤ 0.05 **, ≤ 0.1*)

	Mixed logit model with panel effect	Mixed logit model with panel effect and random coefficients for time and cost	Latent class choice model			
Indicators			LC1 (29%): “It’s my ride”	LC2 (28%): “Sharing is saving”	LC3 (24%): “Time is gold”	LC4 (19%): “Cheap and half empty, please”
*Stated preference attributes*						
Time	−0.318 (−11.05) ***	−0.389 (−10.28) ***	−0.1936 (−3.14) ***	−0.2685 (−5.56) ***	−1.3185 (−3.01) ***	−2.0418 (−2.23) **
Additional time parameter working individuals	−0.0662 (−2.68) ***	−0.0916 (−2.90) ***	N/A	N/A	N/A	N/A
Cost	−1.59 (−17.17) ***	−1.83 (−14.98) ***	−0.6843 (−4.24) ***	−1.1492 (−5.95) ***	−3.0138 (−2.50) **	−15.7452 (−2.03) **
ASC pooled alternative (i.e., pooled and cheaper)	N/A	N/A	−1.7265 (−2.84) ***	2.1580 (4.57) ***	3.0540 (2.99) ***	N/A
1 or 2 extra passengers (dummy)	−0.693 (−2.46) ***	−0.745 (−2.32) **	N/A	N/A	N/A	N/A
2 extra passengers (dummy)	N/A	N/A	−0.3762 (−2.06) **	−0.3762 (−2.06) **	−0.3762 (−2.06) **	N/A
4 extra passengers (dummy)	N/A	N/A	N/A	−0.6818 (−2.37) **	−1.9873 (−1.65) *	N/A
4 extra passengers (per minute in-vehicle time)	−0.0636 (−5.68) ***	−0.0681 (−5.08) ***	−0.0555 (−3.47) ***	N/A	N/A	N/A
Number of passengers (exponential)	N/A	N/A	N/A	N/A	N/A	−0.4661 (−1.89) *
*Random parameters*						
Sigma panel	2.37 (15.19) ***	1.79 (8.01) ***	N/A	N/A	N/A	N/A
Std. dev. in-vehicle time (normally distributed, doubly-truncated z = 1.28)	N/A	0.310 (6.00) ***	N/A	N/A	N/A	N/A
Std. dev. cost (normally distributed, doubly-truncated z = 1.28)	N/A	1.45 (8.84) ***	N/A	N/A	N/A	N/A
*Personal attributes (included in the utility function of the individual alternative)*						
High income	0.880 (2.97) ***	0.840 (2.41) **	N/A	N/A	N/A	N/A
BTM never used	0.522 (2.09) **	N/A	N/A	N/A	N/A	N/A
Frequency bicycle	−0.171 (−2.74) ***	−0.229 (−3.11) ***	N/A	N/A	N/A	N/A
*Model for classes*						
Intercept	N/A	N/A	0.3599 (2.31) **	0.1457 (0.81)	0.0391 (0.19)	−0.5447 (−2.44) **
*Covariates*						
Working individual	N/A	N/A	0.0681 (0.89)	−0.2531 (−2.84) ***	0.1211 (1.20)	0.0640 (0.56)
BTM never used	N/A	N/A	0.2218 (2.93) ***	−0.0363 (−0.38)	−0.0287 (−0.28)	−0.1567 (−1.30)
High personal income	N/A	N/A	0.2625(2.83) ***	−0.0640(−0.51)	0.0424(0.33)	−0.2409(−1.53)
Young individual (18–34 years old)	N/A	N/A	−0.1920(−2.24) **	0.0722(0.74)	0.2244(2.43) **	−0.1046(−0.85)
*Model fit statistics*						
Final log-likelihood	−1594.91	−1572.75	−1533.66			
Adjusted rho-squared	0.281	0.291				
BIC	3262.51	3226.27	3274.73			

*N/A* not applicable/no parameter was estimated

Our second model adds random components to the time and cost attributes, to account for unobserved heterogeneity. Adding a random component to the WTS-related attributes did not improve the model. We tried different distributions for these random components: a normal distribution, a lognormal distribution, and a doubly-truncated (i.e., bounded) normal distribution. The two latter distributions allow to not associate individuals with positive parameter values (which would be counterintuitive for the time and cost attributes). From the three distributions, the doubly-truncated distribution provides the best model fit (truncation is done by normalising the remaining surface). The time-related random component only affects the common time parameter and not the additional time-related parameter concerning working individuals. Unlike in the previous model, not using BTM (bus/tram/metro) did not prove to be significant, and is removed from the final model specification. The final adjusted rho-squared of the two ML models are 0.281 and 0.291 respectively, indicating a better model fit of the second model specification. Both models are estimated using 10,000 Halton draws.

We additionally calculate the values of time (VOT) of the estimated models, which help us further compare their results. The basic VOT calculation (as direct division between the $\beta_{time}$ and $\beta_{cost}$ coefficients), does not apply in the case of random coefficients. In this case, a second order approximation can be used (Seltman 2012). Given that the covariance of both parameters can be assumed to be zero (as a result of the choice model formulation), Frei et al. (2017) approximate the VoT in this case as follows:

1 $$\begin{aligned} VoT & = E\left[ {\frac{{\beta_{time} }}{{\beta_{cost} }}} \right] \approx \frac{{E\left[ {\beta_{time} } \right]}}{{E\left[ {\beta_{cost} } \right]}} - \frac{{Cov\left( {\beta_{time} , \beta_{cost} } \right)}}{{E^{2} \left[ {\beta_{cost} } \right]}} + \frac{{Var\left[ {\beta_{cost} } \right] \times E\left[ {\beta_{time} } \right]}}{{E^{3} \left[ {\beta_{cost} } \right]}} \\ & \approx \frac{{E\left[ {\beta_{time} } \right]}}{{E\left[ {\beta_{cost} } \right]}} + \frac{{Var\left[ {\beta_{cost} } \right] \times E\left[ {\beta_{time} } \right]}}{{E^{3} \left[ {\beta_{cost} } \right]}} \\ \end{aligned}$$

Unlike the model reported in Frei et al. (2017), our time and cost distributions do not follow a normal distribution but rather a doubly truncated normal distribution ($z_{1}$ = −1.28, $z_{2}$ = 1.28). Therefore, the mean remains the same as the non-truncated distribution, but the truncation shrinks the variance of the distribution relative to the non-truncated case. Therefore, the ${\text{Var}}[\beta_{\text{cost}}]$ introduced in (1) has to be adjusted. For our symmetrical case, the corresponding formulation is as follows (we refer the reader to Burkardt (2014) and Johnson et al. (1994) for the general mathematical formulation):

2 $$Var\left[ {\beta_{cost} } \right] = \sigma^{2} \left[ {1 + \frac{{z_{1} \times \emptyset \left( {z_{1} } \right) - z_{2} \times \emptyset \left( {z_{2} } \right) }}{{\Phi \left( {z_{2} } \right) - \Phi \left( {z_{1} } \right)}}} \right]$$

where $\sigma$ is the variance of the non-truncated normal distribution, and $z_{1}$ and $z_{2}$ are the lower and upper truncation bounds of the equivalent standard normal distribution. The functions $\emptyset$ and $\Phi$ are:

3 $$\emptyset \left( z \right) = \frac{1}{{\sqrt {2\pi } }} exp\left( { - \frac{1}{2}z^{2} } \right)$$

4 $$\Phi \left( z \right) = \frac{1}{2} \left( {1 + {\text{erf}}\left( {\frac{z}{\sqrt 2 }} \right)} \right)$$

The WTS calculations are analogous to the VOT ones (including $\beta_{add pax}$ instead of $\beta_{time}$). Values of these VOT and WTS values are depicted in Table 5. As can be observed, values for the ML model with random components are a bit higher. Not capturing the unobserved heterogeneity in the model formulation can thus lead to an underestimation of the VOT and WTS.

**Table 5:** Value of Time (VOT) and Willingness to Share (WTS) values for the estimated models

VOT and WTS values	ML (panel effect)	ML (panel effect and random coefficients)	Latent class choice model			
			LC1: “It’s my ride”	LC2: “Sharing is saving”	LC3: “Time is gold”	LC4: “Cheap and half empty, please”
VOT (€/h)	N/A	N/A	16.98	14.02	26.25	7.78
VOT (non-working individuals) (€/h)	12.00	16.25	N/A	N/A	N/A	N/A
VOT (working individuals) (€/h)	14.50	20.08	N/A	N/A	N/A	N/A
ASC_pooled_alternative/beta_cost	N/A	N/A	2.52	-1.88	-1.01	N/A
WTS 1 additional pass. (€/trip)	0.44	0.52	N/A	N/A	N/A	0.08
WTS 2 additional pass. (€/trip)	0.44	0.52	0.55	0.33	0.12	0.44
WTS 4 additional pass. (€/trip)	N/A	N/A	N/A	0.59	0.66	6.47
WTS 4 additional pass. (€/h)	2.40	2.85	4.87	N/A	N/A	N/A

For the ML model with random components, we obtain a VOT of 16.25 €/h for non-working individuals and 20.08 €/h for working individuals. WTS values are much lower. They amount to 0.52 €/trip when the ride is shared with one or two additional passengers, and 2.85 €/h when the ride is shared with four additional passengers (remember that the ML model included the four co-rider disutility as a time-dependent variable).

Next, we compare the obtained VOT and WTS values with previous studies, in particular those reported in Al-Ayyash et al. (2016) and Lavieri and Bhat (2019). These studies, similarly to this study, include the time, cost and the number of additional passengers as explanatory variables. Al-Ayyash et al. (2016) is set in Beirut, Lebanon, and addresses university students and university employees. It estimates different parameters depending on how often individuals would be willing to adopt the pooled on-demand service for their university commuting habits, and it differentiates between car and public transport commuters. Their obtained VOTs (converted to Euros) range between 3 €/h and 13 €/h. These are lower than our obtained values, which may be arguably attributed to the lower purchasing power of individuals in Lebanon in comparison to those in the Netherlands. Lavieri and Bhat (2019), in turn, is set in the Dallas-Fort Worth Metropolitan Area, USA, and studies commuters. Its obtained VOTs are around 26 €/h for working trip purposes, and 21 €/h for leisure trip purposes, slightly higher values than those found in our study (~ 20 €/h for working individuals).

Regarding the WTS, results from Al-Ayyash et al. (2016) indicate that respondents are willing to pay between 0.5 € and 2 € to perform their ride in a vehicle that allows for a maximum of two extra passengers instead of riding a vehicle that allows for up to five extra passengers. This result resonates well with our findings. In Lavieri and Bhat (2019), the ratio between the parameter of additional passengers and cost yields a disutility of around 0.4–0.8 €/trip per additional passenger. Again, these values are in line with our findings.

We conclude the comparison between the studies comparing the ratio between the WTS and the VOT values in the three studies. The ratios that can be obtained from the different traveller categories analysed in Al-Ayyash et al. lead to values around 0.1. To match their approach and obtain a comparable ratio from our study, we need to consider as WTS value the difference between the four co-rider scenario and the 1–2 co-rider scenario. We obtain ratios of 0.05–0.1 for trips lasting 30–60 min. WTS-VOT ratios in Lavieri & Bhat amount to 0.02–0.07 for the 1–2 co-rider scenario. In this case, our ratios are also in the same range, amounting to around 0.03. This comparison shows that the VOT and WTS values obtained in our study are well aligned with results reported in previous SP experiments.

Finally, we perform the LCCM analysis. We do so with the first ML specification as a starting point. We determine the number of classes to be included in the model based on the BIC (Bayesian Information Criterion) index. The four class model minimises the BIC index and yields a meaningful segmentation, and is therefore adopted. The final model, shown in Table 4, includes different pooling parameters for different classes. This indicates that the sharing attribute is best modelled using different specifications for different individuals. All time and cost parameters are significant at the 95% level and have the expected negative signs. Parameters related to the number of additional passengers are also negative, with a higher disutility the more extra passengers are in the vehicle, as expected. The majority of the passenger related attributes are also significant at the 95% level. Three of the classes include an alternative specific constant (ASC) in their model specification. The positive sign of two of them implies a preference towards the pooled alternative over the individual one when time and cost parameters are zero and there is one extra passenger in the pooled option. A first explanation could be that the two classes prefer sharing their vehicle (e.g., environmental or social considerations). However, individuals in this classes do experience a higher disutility when sharing the vehicle with two individuals than with one, and this is again higher with four individuals than with two (negative related dummy coded parameters, largest for the four extra passenger specification). Therefore, we conclude that the positive ASC is not due to a preference towards sharing the vehicle, but it is linked to the cost-saving characteristic of the pooled alternative. The LCCM also includes four active covariates, which help define the classes and forecast class membership: being a working individual, having a high personal income, never using bus/tram/metro and being aged 18–34. Three of them also played a role in the ML specification, underscoring their relevance in explaining preference heterogeneity in our SP experiment.

**Fig. 3:**
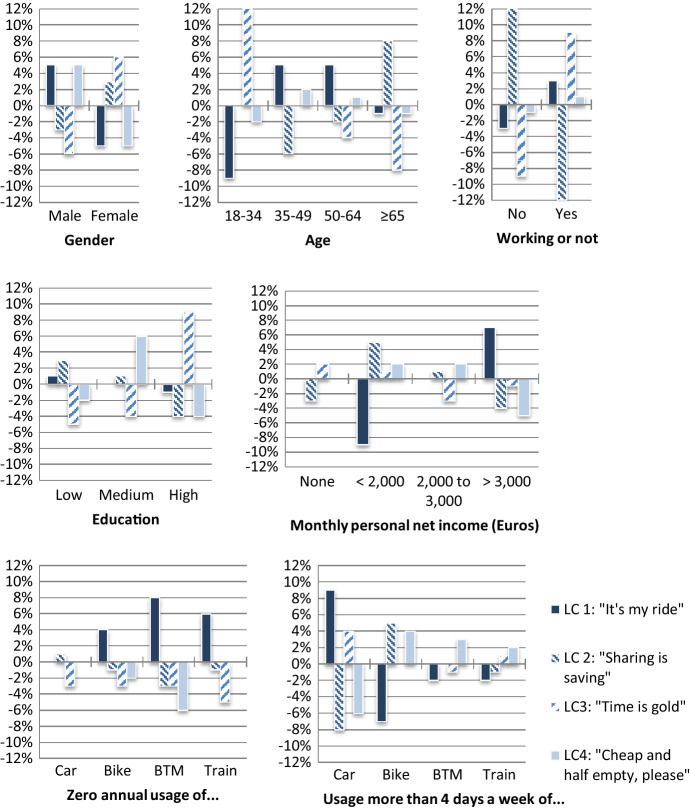
Class profiles regarding different socioeconomic characteristics and mode use frequency (percentage deviations from the estimation sample mean values)

To better understand the main differences between the classes, we calculate the VOT and WTS values for the different classes (Table 5) and depict percentage differences between classes regarding socioeconomic and mode use characteristics (Fig. 3). We also attach a motto to each class, as follows:

- LC 1 (29% of the sample^1^): “It’s my ride”. Individuals in this class experience the highest disutility related to sharing their ride. This preference is confirmed with the attitudinal indicators: this class has the strongest attitude towards privacy, the highest sharing-related time sensitive attitude, and the lowest price sensitive attitude of all classes. “Individual-only” respondents are to be found in this class, amounting to over half of this class’ respondents. Sharing disutility for rides shared with four other passengers is proportional to the in-vehicle time (as specified for the ML model) for individuals in this class. Individuals in the other three classes (less adverse to sharing) perceive it as a per-ride fix disutility. Individuals in this class tend to be male, middle aged (35–64), and have high personal incomes. Regarding current mobility, they differ from the other classes in their higher car usage, and lower bicycle and public transport usage.
- LC 2 (28%): “Sharing is saving”. They are the most positive towards the pooled alternative, which can be explained by their price sensitivity (the pooled option offers them always cheaper rides) and low sharing reluctance. These two characteristics explain why “pooled-only” respondents are to be found (almost exclusively) in this class. Individuals aged 65 and older, females and not working respondents are more predominantly in this class.
- LC 3 (24%): “Time is gold”. These individuals display the highest value of time. They differ from “It’s my ride” individuals in their higher acceptance towards pooling. This higher acceptance explains why despite having a somewhat lower value of time, “it’s my ride” individuals have a more time sensitive attitude towards increases in time caused by sharing their ride. Their strong time sensitivity, together with the little disutility they attach to pooling per se cause the ASC of this class to have a positive sign. Note, however, that the lowest added time for the pooled alternative is three minutes, and “Time is gold” individuals already associate a larger disutility towards pooling for the three minutes extra time than the positive utility stemming from the ASC, implying that if no cost differences would exist, the individual alternative is preferred for the scenarios included in the SP. Respondents also seem to be more time sensitive for shorter trips (i.e., for the ≤ 12 km version of the SP experiment), with 55% of individuals in this class having had the short version, versus 45–50% in the other three classes. Young (18–34), female, highly educated individuals characterise this class. Frequent car usage in this class is also higher than the average, second to “It’s my ride” individuals.
- LC 4 (19%): “Cheap and half empty, please”. This is a very cost sensitive class, with a value of time even lower than the “Sharing is caring” class. The main difference compared to the second class is the more negative preference of “Cheap and half empty, please” individuals towards the pooled alternative, especially when four extra passengers are in the vehicle (the disutility regarding pooling with an increasing number of passengers increases exponentially). This explains why, despite their lower value of time, “Cheap and half empty, please” did trade between the individual and the pooled alternative in the SP experiment. This fourth class has a higher share of male and middle educated respondents than the average sample. The likelihood to belonging to this class is similar for individuals with different age groups or working situation.

We now turn to validating the obtained models by comparing the prediction rate of both the estimation and the validation subsamples (all models were estimated on 80% of the sample and the remaining 20% was kept for validations purposes). We obtain respectively 71% and 71% for the in-sample data and 73% and 72% for the out-of-sample data for the two ML models. Both offer adequate and similar prediction performance. We obtain similar prediction rates (72% and 75% for the estimation and validation samples respectively) for the LCCM using prior membership probabilities (i.e., using only information regarding the active covariates to infer the membership probabilistic distribution to each of the classes). Moreover, when using the individual’s posterior membership probabilities of the LCCM (i.e., statistical inference using an empirical Bayes method which includes information from the observed choices and not exclusively the active covariates to determine the individual’s probabilistic distribution to each of the classes), a 93% correct prediction rate for both estimation and validation samples is achieved. This, in turn, suggests that the presented classes succeed in describing the existent heterogeneity of different individuals regarding preferences towards time, cost and pooling attributes when choosing between individual and pooled on-demand services.

### Scenario analysis

We subsequently perform a scenario analysis as model application. These scenarios seek to quantify the impact of time, cost and the number of passengers on the willingness to request pooled rides (over individual ones). The scenarios are designed to demonstrate the impact of the modelling results and, thus, understand their policy implications. Consequently, scenario analyses can help prioritise effective policies triggered by the behavioural change caused by new mobility services (Tarabay and Abou-Zeid 2019). The ML model with random components has the lowest BIC value out of the three previously estimated models (3226.27 vs. 3262.51 and 3274.73), showing the best model fit, and is therefore the one used for the scenario analysis. The discrete choice model is based on disaggregate demand, but the aggregate demand is necessary to derive indicators at the population level, i.e. we need to weight individuals such that they mirror the real distribution of the population. Even if the socioeconomic characteristics of our sample are already quite representative, we weight our sample to mirror the age and gender shares of the target (urban) population for the scenario analysis.

**Fig. 4:**
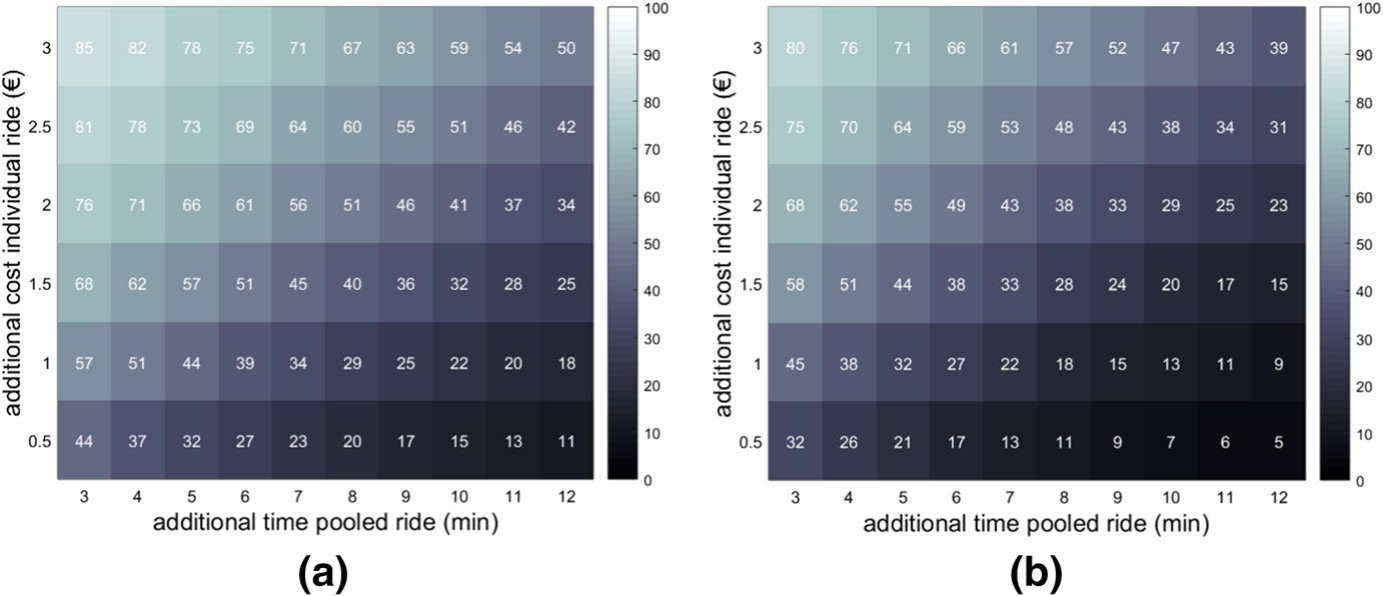
Shares for the pooled alternative for different time–cost trade-offs for the **a** 1 or 2 extra passenger scenario, and **b** 4 extra passenger scenario. Duration individual trip: 20 min

Figure 4 shows the effect of varying time–cost trade-offs in the expected percentage of requested pooled rides for the one or two extra passenger scenario and the four extra passenger scenario (versus the individual shares). As expected, the pooled share increases with increasing price difference and decreasing time difference. For the same time–cost trade-off, the achieved pooled share with one or two additional passengers is 5–13% higher than with four additional passengers (mean 10.5%, median 11.1%). Pooled shares vary in our scenarios from 5 to 85%, showing the great impact that the studied range of additional times and fees has on the outcomes. In our experiment, the pooled alternative is preferred by 85% of the individuals when this entails a €3.00 price reduction and only 3 min of extra time with either one or two additional passengers.

For the base scenario (20 min and €6.00 for the individual ride; + 7 min and −€2.00 for the pooled ride), we obtain the following shares: 56% for the pooled alternative if sharing with one or two extra passengers and 43% in case of sharing with four extra passengers. These shares are well above current shares for pooled rides reported from deployed commercial on-demand services (reported in the Introduction). We highlight three main reasons for that. First, it can be that real time–cost trade-offs presented to on-demand users are more negative for the pooled alternative than those represented in our base scenario. Second, our results are inevitably influenced by the attribute levels used in our SP design. And third, while some segments of the population are overrepresented among users of on-demand services, our scenarios make the breakdown between individual and pooled services for a representative sample of the overall population. For example, currently a higher percentage of higher income individuals tend to use on-demand services. Their preference towards the individual alternative (confirmed by our estimated model), explains the lower pooled share in reality compared to the one found in our scenario. Still, these results suggest that there is potential for the share of pooled requests to increase once on-demand services as a whole become more common place.

**Fig. 5:**
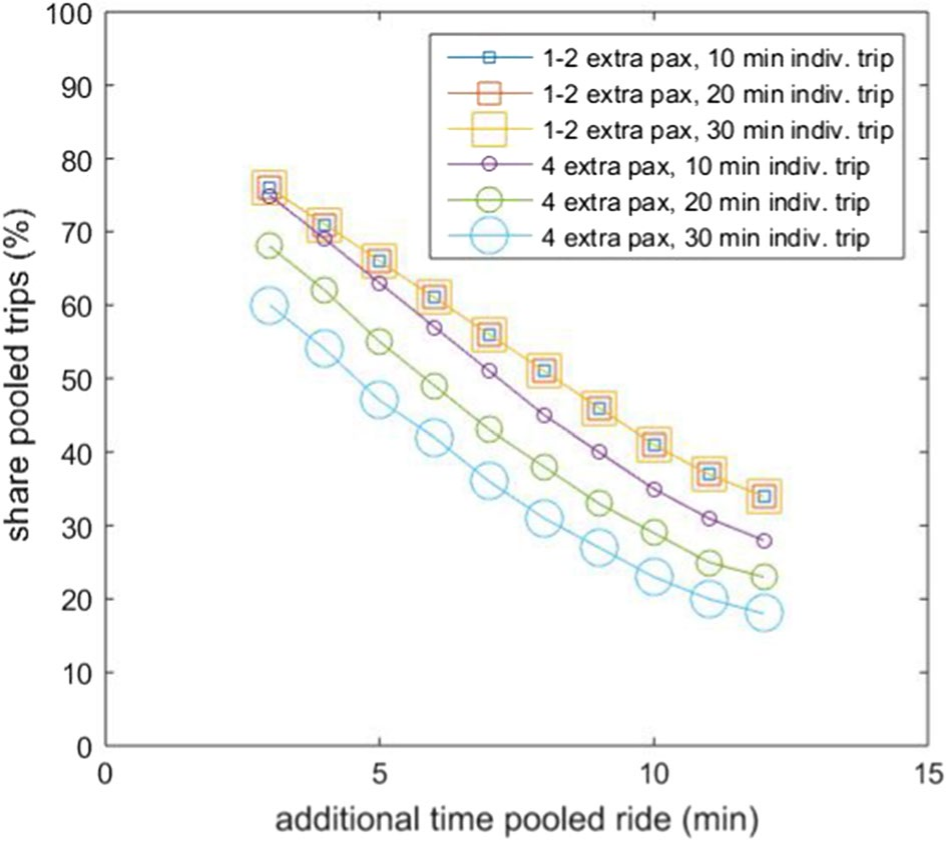
Influence of varying time loss (in the pooled alterative) with varying individual trip time durations on the shares for the pooled alternative. Extra cost of individual trip in the shown scenarios: + €2.00

**Fig. 6:**
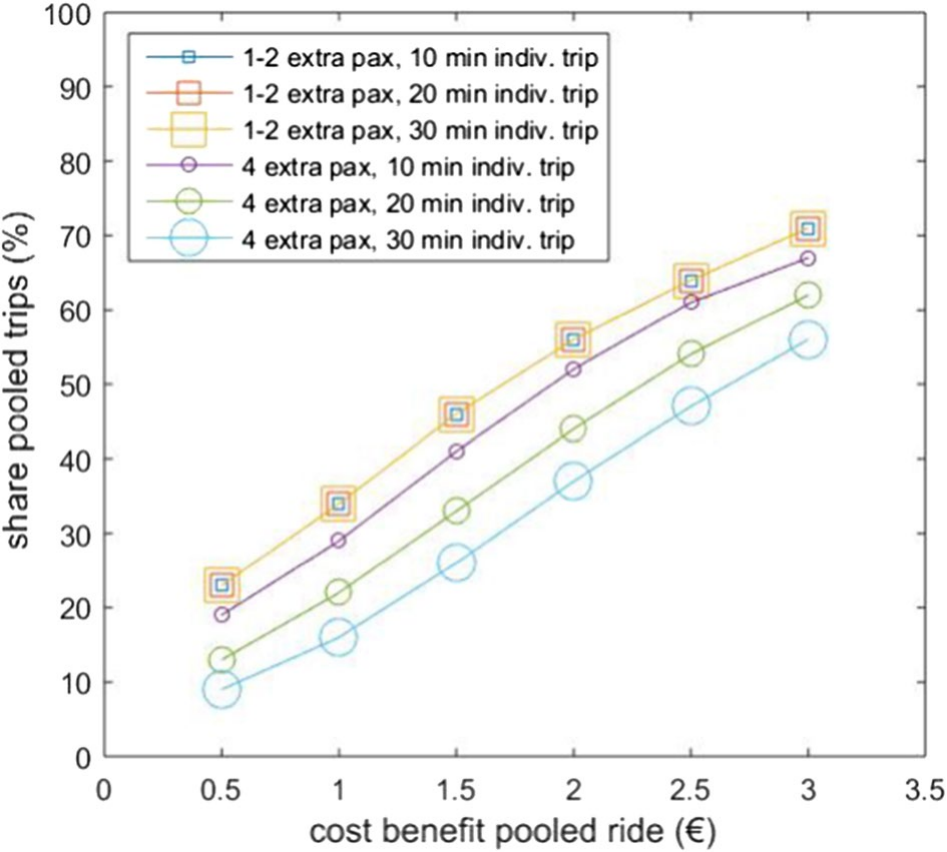
Influence of varying cost gains (in the pooled alterative) with varying individual trip time durations on the shares for the pooled alternative. Extra time of pooled trip in shown scenarios: + 7 min

In our model formulation, only absolute differences in time and cost between the two alternatives matter (given that an additional minute or Euro are associated with the same linear disutility in the individual and pooled alternatives). For the four passenger scenario, however, sharing disutility varies as a function of the total time of the pooled ride. Figures 5 and 6 show the influence of time and cost, respectively, on the share of pooled trips (while keeping the other variable constant). For any 10 min of added individual ride, we see a drop of around 7% in the share of individuals who opt for the pooled alternative when the trip is shared with four additional passengers. This does not affect the shares when sharing rides with just one or two extra passengers, given that, in the ML model, the one and two co-rider disutility (unlike the four co-rider disutility) is a per trip and not a per minute value.

## Discussion

In this section, we further discuss the study results. We divide it in two parts. First, we elaborate on implications and recommendations that stem directly from the study results; and then, we further discuss modal shifts that can be offset by pooled on-demand services, as well as the importance of framing in the uptake of these services.

### Implications and recommendations

The model estimation results (more tangible thanks to the scenario analysis) underpin that the percentage of pooled rides greatly depends on the time–cost trade-off. They also show that the disutility stemming from sharing per se is less of a deterrent than the time–cost trade-off in determining the likelihood to choose pooled rides, in line with previous research (Lavieri and Bhat 2019; Sarriera et al. 2017; Stoiber et al. 2019). When pooling rides, the disutility of having one or two extra passengers is constant, regardless of the trip length. This disutility further increases in the event that one shares the ride with four additional passengers, in which case it increases the longer the pooled trip. Our study suggests that this may constitute a tipping point in the way that the sharing disutility is perceived. This finding can also be due to the respondents’ perception that a vehicle larger than a normal car is needed in the four co-rider situation. Further research is needed to delve into the source of the difference in disutility. Regardless of the underlying cause, most of the pooled requests (for which a matching trip is found) are in reality currently shared with just one additional passenger (Chen et al. 2018), and simulation studies suggest that at most two or three requests in a vehicle (i.e., one or two additional passengers) would be the rule for the majority of the pooled on-demand rides (Bischoff et al. 2017; ITF 2016). Offering rides with the upfront information that only one or two additional passengers will be in the vehicle can increase the share of pooled requests. Moreover, this would not imply high losses in terms of fleet utilisation efficiency given that having four passengers in the same vehicle would have been a rare occurrence anyhow.

The latent class analysis identifies four distinct classes that explain taste variation. These classes have different specifications to represent WTS, indicating that the disutility attributed to sharing is perceived differently among individuals. Individuals in “It’s my ride” class (29% of the sample), attribute a high penalty to sharing (high WTS value) and have high a high value of time (VOT). As a result, they strongly prefer individual rides. Individuals in this class have different travel patterns than those in the other classes, with a higher car usage, and a lower bicycle and public transport usage. Previous studies also found that adopters of new shared mobility alternatives tend to cycle more and perform fewer private car trips (Kopp et al. 2015). Individuals in the “Cheap and half empty, please” class (19%), also experience a high WTS penalty, but only when the ride is shared with four additional passengers. The remaining two classes show a low WTS penalty, and have either time (“Time is gold”, 24%) or cost (“Sharing is saving”, 28%) as the main determinant of their choices. The somewhat higher shares of females in these two classes suggests a lower WTP penalty for this population segment.

Policymakers may be interested in avoiding scenarios with high individual on-demand shares, and make riders internalise the externalities associated with the increase in VMT associated with these rides. Introducing a per-ride tax on individual requests (or a per-passenger subsidy for pooled rides) could be a policy measure to do so. In fact, different cities in the US have already implemented a tax on on-demand rides (Hu 2018). In the case of one or two extra passengers in our base scenario, an extra €1.00 individual tax (or alternatively, pooled subsidy), would raise the percentage of individuals who prefer the pooled alternative from 56 to 71% (i.e., + 15%).

Regarding the additional incurred time, simulation studies suggest that a time disutility in pooled rides of just 3 min per passenger are possible (Alonso-Mora et al. 2017; Bischoff et al. 2017), yet real data indicates that currently the mean lies around 10 additional minutes per passenger (Li et al. 2019). A reduction from 10 to 3 min in incurred additional time would imply, in our simulated scenarios, an increase from 41% of individuals preferring pooled rides to 76% (+ 35%) (assuming €2.00 price difference and one or two extra passengers). A higher demand density of such rides may be necessary to reduce the extra time. Allocating curb space for pooled on-demand rides can both speed the pick-up/drop-off process and ensure that there is clarity regarding the exact pick-up point, reducing the additional incurred time.

### Further considerations

The positive benefits of pooled rides may be, to some extent, offset by modal shifts from transit and active modes. Our modelling results indicate that it is individuals with a higher usage of cycling and transit that are more attracted to the pooled alternative. Indeed, previous research indicates modal shifts of 34–54% from transit and (to a lesser extent) active modes to on-demand services (Gehrke et al. 2018; Henao and Marshall 2018; Rayle et al. 2016; Tirachini and Gomez-Lobo 2019), while higher percentages 48–63% have been found in a study considering pooled services exclusively (Chen et al. 2018). Competition with cycling stems from the short distance of many of the on-demand trips. For example, average distance of the on-demand rides in the city of Chengdu are eight kilometres (Li et al. 2019), the distribution being right-skewed (i.e., the median is lower than the mean). This suggests that there is a significant share of trips within cycling distance. The competition between transit and pooled on-demand services in particular can be best explained by the fact that these pooled services present a cost-effective option for some otherwise underserved origin–destination pairs (Alonso-González et al. 2018; Schwieterman 2019), or provide a premium service for certain segments of transit trips. Cooperation between transit authorities and on-demand companies can help improve the overall provided urban mobility (e.g., by improving the level of service of traditional transit for the more dense corridors and facilitating the usage of pooled on-demand services for the first-last mile leg).

An additional remark should be made explicit regarding this research. Our SP experiment was the last part of a longer survey in which only pooled on-demand services were considered. The individual option was introduced to respondents only at the end of the survey. Individuals can therefore see the individual option as a “service upgrade”, while major on-demand providers offer the individual ride as their base product and the pooled option as their cost-saving one. Framing influences individuals to choose the default option, and previous research has found that framing can influence travel-related choices (e.g., Avineri and Owen 2013; Garcia-Sierra et al. 2015; Mattauch et al. 2014). We speculate that the estimated pooled shares would have been somewhat lower if respondents had considered the individual service as the base and the pooled option as a “service downgrade”.

## Conclusions

This paper has analysed individuals’ willingness to share rides by comparing individuals’ preferences towards individual versus pooled alternatives. Previous studies have shown the potential of pooled services to tackle current urban challenges, yet currently the large majority of on-demand trips are requested as individual trips. To help explain the low pooled shares, we first designed a stated preference (SP) experiment targeting individuals living in (sub)urban areas of the Netherlands. We performed a choice modelling analysis that accounted for the unobserved heterogeneity of individuals, including mixed logit and latent class choice models in our analysis. Additionally, this research applied the estimated behavioural modelling results in a scenario analysis. These scenarios simulated the impact of different time–cost trade-offs, as well as the impact of individuals’ disutility associated with sharing the rides with different numbers of extra passengers.

Results show that the share of individuals who prefer to share rides lies primarily on the time–cost trade-off they encounter, rather than on the on-board discomfort associated with ride-pooling. There are large differences between the extra time that individuals pooling rides currently experience and the extra time that simulation studies believe to be possible. Also, price discounts associated with pooled rides are often insufficient due to the low probability of matching rides. This may explain the low shares of shared rides currently observed in practice, and suggest a potential vicious cycle unless targeted efforts to generate a positive spiral are made. In that case, our results suggest potential to achieve an increase in the share of rides that are booked as shared. Given the expected large gains in VMT from pooled rides reported in literature, policy makers could consider imposing a tax on individual rides in order to internalise the related externalities and steer demand towards pooled alternatives instead.

Our obtained behavioural model also indicates that current travel patterns and personal income influence individuals’ preferences to share rides. Less than one third of individuals (“It’s my ride” individuals) have strong preferences towards individual rides, and these individuals are characterised by a more unimodal car behaviour. This suggests that: (1) the uptake of pooled rides can still increase considerably and (2) current car-centred individuals are less likely to shift to more collective modes of transport. Our findings also indicate that the willingness to share of individuals depends on the number of additional passengers. Therefore, a beforehand specification of the number of people that are expected in the pooled ride (or a prediction thereof) can encourage individuals to use the pooled alternative. In the absence of such a prediction, users may refrain from opting for the pooled service in order to avoid the most adverse case in which they share their ride with four or more co-riders.

Finally, we need to acknowledge the limitations associated with the present study. Other than those related to the stated preference nature of the data, we pinpoint two main limitations. First, our research considers the choice between an individual and a pooled on-demand alternative. In reality, individuals have the option to opt out and perform the trip using a different transport mode. Individuals who opt in may not mirror a representative sample of the population, and, thus, the split between both alternatives may differ from the obtained values in this study. Future research could include the parameters estimated in this research to calculate market shares, calibrating the model with the breakdown that they observe in their specific setting. Second, previous studies have identified trust as an important aspect when pooling rides (Amirkiaee and Evangelopoulos 2018). This suggest that individuals’ willingness to pool rides may depend on their previous experience with the service (as well as of those around them), which is not contemplated in this study. Future research could study the willingness to share and the time–cost trade-offs manifested by users of platforms that already offer both individual and pooled services, and investigate how (or if) these aspects change with individuals’ experience. Future research could also delve into how vehicle size or uncertainties in the number of passengers affects the obtained willingness to share for pooled on-demand trips.

## Exploratory factor analysis of the attitudinal indicators

See Table

**Table 6:** EFA loadings, mean and standard deviation of the attitudinal indicators and significance of independent t-tests between traders and each of the non-trading groups (equal variance not assumed.) (Legend: “individuals-only” vs “traders” p ≤ 0.01 **, p ≤ 0.05 *; “pooled-only” vs “traders” p ≤ 0.01 (+ +), p ≤ 0.05 ( +).)

Attitudinal statement (and source where applicable)	EFA loadings (pattern matrix)	Mean (sd) of total sample	Mean (sd) of “individual-only”/ “trading”/ “pooled-only” respondents	t-test signific. (2-tailed)
*Privacy attitude*				
It makes me uncomfortable to ride with strangers on public transport (modified from (Rubin 2011))	0.622	2.31 (0.90)	2.67/2.26/2.13 (0.98/0.88/0.84)	** ()
I think the public transport is not so clean or decent	0.571	3.06 (0.93)	3.31/3.06/2.86 (0.96/0.91/0.94)	** ( +)
I like the privacy in the car or bike (modified from (Spears et al. 2013))	0.438	3.76 (0.87)	4.07/3.74/3.53 (0.79/0.85/0.97)	** ( +)
People like me only use their own bike and/or car	0.407	3.08 (1.13)	3.41/3.03/3.01 (1.12/1.12/1.13)	** ()
*Cost sensitivity and multimodal mind-set **				
I would use the car less if there would be a cheaper alternative	0.602	3.29 (1.05)	2.95/3.31/3.53 (1.06/1.03/1.00)	** ( +)
I choose to travel with public transport or to share rides to reduce my trip costs	0.583	3.30 (0.98)	2.64/3.37/3.61 (1.03/0.91/0.93)	** (+ +)
I am willing to try new ways to travel	0.534	3.46 (0.83)	3.14/3.51/3.55 (0.99/0.79/0.83)	** ()
I often compare different travel options and transport modes before choosing how to travel (modified from (Atasoy et al. 2010))	0.500	2.78 (1.04)	2.56/2.81/2.88 (1.12/1.03/0.99)	* ()
I do not mind which transport mode I use, as long as it suits my trip needs	0.401	3.44 (1.01)	3.14/3.48/3.53 (1.15/0.98/0.94)	** ()
*In-vehicle time flexibility attitude*				
I would not mind if other travellers get in or off the FLEXI vehicle during my ride (reversed) (modified from (Al-Ayyash et al. 2016))	0.674	2.50 (0.96)	3.13/2.43/2.23 (1.07/0.89/0.89)	** ( +)
I would find it annoying that FLEXI does not drive the fastest route (e.g., FLEXI’s route is 18 min instead of 15 min) (modified from (Al-Ayyash et al. 2016))	0.578	2.91 (0.96)	3.27/2.88/2.66 (1.08/0.91/0.98)	** (+ +)

*We cover multimodality together with cost sensitivity since previous research found that cost sensitive individuals tend to also be more interested in shared modes (Burkhardt and Millard-Ball 2006)

6.

## Footnotes

1 Note that latent class models allocate individuals to classes in a probabilistic and not in a deterministic manner. An individual could, for example, belong to classes one to four with weights 0.5, 0.3, 0.1 and 0.1, respectively (the sum always amounts to one and an individual can have the same probability to belonging to different classes). All percentages regarding class size or class profile mentioned here refer to the sum of these probabilistic distributions of individuals.
